# Profiles of Career Indecision: A Person-Centered Approach with Italian Late Adolescents

**DOI:** 10.3390/ejihpe14050095

**Published:** 2024-05-15

**Authors:** Anna Parola, Jenny Marcionetti

**Affiliations:** 1 Department of Humanities, University of Naples Federico II, 80133 Naples, Italy; 2 Department of Education and Learning, University of Applied Sciences and Arts of Southern Switzerland, 6600 Locarno, Switzerland; jenny.marcionetti@supsi.ch

**Keywords:** career decision-making difficulties, latent profile analysis, late adolescents

## Abstract

Choosing career paths in today’s contemporary labor market complexity is becoming more and more challenging for adolescents and young people. Career indecision could take over, and assessing its facets could guide career interventions to support the career decision-making process. To create increasingly tailored career guidance interventions, using a person-centered approach, this study aimed to understand whether profiles of late adolescents based on their career decision-making difficulties might be circumscribed. A total of 776 Italian late adolescents took part in this study. The assessment of career decision-making difficulties was conducted through the Career Decision-Making Questionnaire (CDDQ). To determine the optimal number of profiles, a Latent Profile Analysis (LPA) using the stepwise approach was used. Moreover, a multinomial logistic regression was conducted to study whether school grade and sex predicted profile membership. LPA revealed a four-profile model: “Lower Indecision” (Profile 1, 39%), “High Indecision” (Profile 2, 23%), “Very High Indecision” (Profile 3, 7%) and “Moderate Indecision” (Profile 4, 31%). Being enrolled in the last year of high school significantly predicted belonging to Profile 2 and Profile 3. Practical implications were discussed in light of these findings.

## 1. Introduction

Throughout adolescence and emerging adulthood, individuals encounter significant challenges when making career decisions, which is a notably demanding developmental task [1,2]. Despite the inevitability of this process, it often proves challenging and may lead to difficulties or suboptimal outcomes [3,4,5]. The repercussions of making inappropriate career decisions extend to various aspects of life, including career environments and relationships with significant others, making career decision making a potentially stressful process.

Addressing these challenges serves as a beneficial starting point for potential career guidance interventions [6]. Furthermore, the complexities of the current world of work, characterized by constant change, calls for multi-faceted and unstable career paths [2,7]. Aligned with Levin and colleagues [8], identifying typologies of career indecision enhances understanding of the career decision-making process and facilitates screening and career guidance interventions assisting individuals in developing decision-making skills.

Accordingly, this study aims to identify career indecision profiles using data from a sample of Italian late adolescents and assess whether sex and school grade can influence the likelihood of being classified into a specific profile.

### 1.1. Theoretical Framework

Career indecision encompasses the challenges individuals face in the process of making career decisions [1]. Gati et al. [1] introduced a hierarchical taxonomy following the Decision Theory framework [9,10,11,12], delineating potential problems and difficulties individuals encounter in this process.

According to the Gati taxonomy [1], these difficulties can be classified into different categories according to their timing (before or during career decision-making process), cognitive or affective source, impact (hindering an individual’s ability to make a choice or resulting in a decision that is less than optimal), and the type of intervention required to assist the individual. The first major category, Lack of Readiness, pertains to the period before the decision-making process, where individuals perceive themselves as unable to initiate the process. This category includes (a) lack of motivation, i.e., unwillingness to decide at this specific time; (b) general indecisiveness, i.e., unwillingness to make decisions in general; and (c) dysfunctional beliefs, i.e., distorted perception of career choices and their consequences. The second major category, Lack of Information, pertains to individual’s perception of not having sufficient information necessary for career path selection. The category encompasses (a) lack of information about the process, i.e., a lack of knowledge concerning the stages involved in the career decision-making process; (b) lack of information about the self, i.e., the perception of inadequate information about oneself; (c) lack of information about the occupations, i.e., lack of information on possible professions; and (d) lack of information on the ways of obtaining additional information, i.e., lack of information on ways to obtain additional information or how to get help to ease the process of making career decisions. Finally, the third category, Inconsistent Information, refers (as the previous category) to the moment in which the career decision-making is in progress, where individuals may perceive inconsistencies in the information available regarding professional path. This category includes (a) unreliable information, a sense of contradictory information related to oneself or the occupations taken into consideration; (b) internal conflicts, a state of inner confusion determined by the fact that aspects considered necessary by the individual for the professional choice are incompatible among them; and (c) external conflicts, i.e., a gap between individual professional preferences and suggestions given by other significant people.

Gati et al. [1] devised the Career Decision-Making Questionnaire (CDDQ) for the assessment of these ten dimensions of decision-making difficulties, which is widely used in studying decision-making processes, predictors, outcomes and career counseling screening assessments [13,14]. Several studies have confirmed the psychometric validity and reliability of the measure across various contexts (for example, Refs. [3,15,16]). Indeed, the CDDQ has been translated into more than 50 languages and adopted in more than 60 countries, always proving to be a sound and robust instrument from a psychometric point of view and with good reliability and validity. According to Xu and Bhang [17], the CDDQ is one of the three reliable and valid measures of career indecision currently in use (in addition to the Emotional and Personality Career Difficulty Questionnaire (EPCD; Ref. [18]) and the Career Indecision Profile [19]. Furthermore, the CDDQ has been shown to be one of the most useful tools in the hands of career practitioners: Gati et al. [20] found good agreement between career practitioners’ opinions and clients’ responses on the CDDQ.

Regarding the variables involved in the process, many studies have addressed the individual variables linked to career decision making, including personality traits [21,22,23], self-perceptions [24], self-evaluation [25], and emotional intelligence [21]. Some studies indicate that the career decision-making process could be more influenced by context [26,27,28] or culture than individual dispositions and tendencies [29,30].

Furthermore, several studies indicate that age and school level may influence career indecision [31,32,33]. While Albion and Fogarty [34] suggest that career decision-making difficulties tend to decrease from adolescence to early adulthood, Parola and Marcionetti [28] propose that career indecision increases approaching career transition turning points. Regarding the role of sex, findings are controversial. While many studies report no sex differences in the career decision-making process [1,31,35,36,37,38,39], others highlight differences in lack of motivation [4,40] and external conflicts [4,40,41], with males experiencing more significant difficulties than females.

In a recent study, Levin and colleagues [42] examined 32.556 individuals across various countries. They found that individuals aged 19–24 manifested the greatest difficulties, which was followed by those aged 14–18, and 25–30. Additionally, males reported higher scores than females in all three career decision-making difficulties and total score, although the effect size was reported to be very small.

### 1.2. Current Study

Levin and colleagues [8] pointed out inconsistencies in clustering studies on career indecision. Synthesizing previous research (Ref. [8], Supplementary Materials A), they highlighted three common types: (a) individuals with a developed vocational identity but little commitment to a career choice; (b) those with poorly developed vocational identity and little commitment; and (c) individuals with a decided career path and well-developed vocational identity. However, many studies are limited by conflating causes and consequences of career indecision, relying on traditional cluster analysis, or extensive batteries of measures. To address these limitations, Levin [8] developed typologies of career indecision based on a single assessment using the CDDQ, finding five profiles: Unmotivated, Indecisive, Unrealistic, Uninformed, and Conflicted. Unmotivated refers to individuals who are insufficiently motivated to finalize their career choice. Indecisive includes individuals who commonly experience challenges in making decisions. Unrealistic comprises individuals having dysfunctional beliefs. Uninformed refers to individuals with a high lack of information. Finally, Conflicted is related to external conflicts, while internal conflicts and the presence of unreliable information compounded the issue.

In the most recent study, Levin and colleagues [43] investigated a typology of career indecision across populations from 16 countries, identifying three types not previously reported by previous results [8]: (a) the occupations-uninformed, (b) externally conflicted, and (c) internally conflicted types. Notably, this study did not include an Italian sample.

The aim of this study is to pinpoint subpopulations among Italian late adolescents using the CDDQ. The study of career indecision profiles requires an in-depth analysis of the context. National institutional differences, particularly in relation to the labor market entry process, significantly influence youth career decision-making [44]. In Italy, high school tracks vary in purposes and curricula, with technical and vocational tracks providing specific skills for sectors like business and technology, and academic/general tracks preparing students for university. Students have the freedom to choose their university faculty regardless of their high school track. In the Italian context, the most challenging decision appears to be the one made after high school, as individuals are confronted with the choice of continuing their education, seeking employment, or pursuing both paths [28].

Using a person-centered approach is strategic given the increasing individualization of the career process [45,46], allowing for a nuanced understanding of career indecision profiles and their predictors and outcomes. Whereas the variable-centered approach assumes that career indecision manifests itself in the same way across a population of late adolescents, the person-centered approach leaves open the possibility that a career indecision might be manifested differently depending on the strength of the career indecision. Furthermore, the person-centered approach allowed to grasp profiles that can differ quantitatively and qualitatively [45]. Quantitative profile refers to the level of the career decision-making difficulties across groups of individuals, while qualitative differences refer to the shape of the profile or the relative level of the indicators within a group [47]. The identification of types of individuals with distinct combinations of causes of career indecision in the adolescent’s population enhance the understanding of the adolescent difficulties (types and prevalence).

Thus, this study aims to develop career indecision profiles applicable to research and career guidance. Although research on career decision-making difficulties has taken a person-centered approach, overall, findings are still limited. In addition, no study has used an Italian sample. Context plays a crucial role in determining the difficulties that adolescents and young adults may encounter in the decision-making process. The current study explored the heterogeneity of individuals’ career decision-making difficulties, focusing on late adolescents, in order to fill these gaps in the literature. In turn, the results may provide useful information in the field of career guidance in Italy.

## 2. Methods

### 2.1. Participants and Procedure of Data Collection

A total of 776 late adolescents with a mean age of 18.4 (SD = 0.87), 380 males and 396 females, participated in this study. Participants were recruited through University–school collaborative networks, involving five different high schools in Campania, southern Italy (2 in Naples, 1 in Benevento, 1 in Avellino, and 2 in Salerno), with students from the fourth (n = 422) and fifth (n = 354) grades. Prior to data collection, permission to administer anonymous self-report questionnaires was obtained from the high school principals. For participants under 18 years old, parental consent was obtained. The questionnaires were completed by the students in a classroom under the supervision of the first author with professors excused from the room. Participants were informed about the extent of the study, assured of the confidentiality of their responses and given the option to withdraw from the research at any time. The study obtained approval from the Ethical Committee of the University of Naples Federico II (code: 20/2019) to ensure participant protection.

### 2.2. Measures

The Career Decision-Making Questionnaire [1] validated in Italian by Di Fabio and Palazzeschi [48] was used. The measure comprises 34 items rated on a nine-point Likert scale, ranging from 1 (“does not describes me”) to 9 (“describes me well”). The questionnaire assesses ten dimensions considered as causes of career indecision: Lack of motivation (example of item: “I believe that I do not have to choose a career now because time will lead me to the right career choice”), General indecisiveness (example of item: “It is usually difficult for me to make decisions”), Dysfunctional beliefs (example of item: “I expect that entering the career I choose will also solve my personal problems”), Lack of information about the process (example of item: “I find it difficult to make a career decision because I do not know what factors to take into consideration”), Lack of information about the self (example of item: “I find it difficult to make a career decision because I do not know what my abilities and/or personality traits will be like in the future”), Lack of information about the occupations (example of item: “I find it difficult to make a career decision because I don’t know what careers will look like in the future”), Lack of information on the ways of obtaining additional information (example of item: “I find it difficult to make a career decision because I do not know how to obtain additional information about myself”), Unreliable information (example of item: “I find it difficult to make a career decision because I have contradictory data about the existence or the characteristics of a particular occupation or training program”), Internal conflicts (example of item: “I find it difficult to make a career decision because my skills and abilities do not match those required by the occupation I am interested in”), and External conflicts (example of item: “I find it difficult to make a career decision because people who are important to me do not agree with the career options I am considering and/or the career characteristics I desire). The scores for each category are calculated as the mean of the items within that category with higher scores indicating greater career indecisiveness.

Cronbach’s alphas in this study were 0.75 for Lack of motivation, 0.75 for General indecisiveness, 0.72 for Dysfunctional beliefs, 0.88 for Lack of information about the process, 0.85 for Lack of information about the self, 0.80 Lack of information about the occupations, 0.81 for Lack of information on the ways of obtaining additional information, 0.75 for Unreliable information, 0.75 for Internal conflicts, and 0.75 for External conflicts.

### 2.3. Statistical Analysis

Preliminary analyses, including means, standard deviations, correlation analysis between study variables, skewness, and kurtosis, were conducted. Additionally, the assumption of missingness at random for missing values was assessed using the MCAR test [49]. The results indicated that the percentage of missing data did not exceed 5%, and Little’s test was not significant (χ^2^ = 11.535, *df* = 17, *p* = 0.827), supporting the assumption that the missing values were missing completely at random. Therefore, missing data were handled using the full-information maximum-likelihood method (FIML) [50].

In the first step, confirmatory factor analysis (CFA) was performed to test the factorial structural model of the CDDQ for this sample. The robust maximum likelihood (MLM) estimator was used as an estimator [51,52,53]. To assess the model fit, the Satorra–Bentler chi-squared test (S–Bχ^2^), the comparative fit index (CFI), the root mean square error of approximation (RMSEA), and the standardized root mean square residual (SRMSR) were used. To evaluate the goodness of fit, the following cut-off criteria were chosen: a CFI higher than 0.90, an RMSEA lower than 0.08, an SRMR lower than 0.08, and a χ^2^/df ratio value of 3 or less [51,52,53].

In a second step, using a person-centered approach, a latent profile analysis (LPA) with a robust maximum likelihood estimator (MLR) was performed to identity profiles of career indecision. LPA is a recommended technique for exploring types of individuals as a person-centered approach [54]. The LPA is a probabilistic and model-based technique [54], and its advantage over other cluster analytic methods lies in the ability to classify individuals into specific profiles based on membership probabilities directly estimated from the model as well as the possibility of using different kinds of variables and including covariates in the model [55]. The optimal number of profiles was determined using a stepwise approach, beginning with two profiles and incrementally increasing the number of latent classes until convergence problems arose or the fit information criteria suggested that additional classes were unlikely to yield valid results [56]. Fit information criteria including the Akaike information criterion (AIC; Ref. [57]) and the Bayesian information criterion (BIC; Ref. [58]) were examined with lower values indicating better fitting models [59]. Parsimony of classes was assessed using the Lo–Mendell–Rubin adjusted likelihood ratio test with *p* > 0.05 (LRT; Ref. [60]) and the Bootstrapped likelihood ration test with *p* > 0.05 (BLRT; Ref. [61]). The entropy statistic was also used to evaluate model-based classification accuracy with values between 0.60 and 0.80 considered acceptable [62,63]. Additionally, average posterior probabilities were examined to assess the accuracy of individual classification into their most likely class with higher probabilities close to 1 indicating greater confidence in class membership. Finally, an R3STEP command for multinomial logistic regression [64] was employed to investigate whether sex and school grade predicted profile membership. The R3STEP method employs multinomial logistic regression analysis to predict the likelihood of belonging to a profile with antecedent variable values. This method boasts several advantages. It can account for measurement error in the most likely profile variable. Moreover, it considers the varying probabilities of belonging to profiles, and the antecedent variables analyzed do not affect the content of profile solutions [64].

## 3. Results

The mean, standard deviation, skewness, and kurtosis for each cause of career indecision are displayed in Table 1. Table 2 presented correlations among the ten sources of career indecision, sex, and school grade. Overall, sex (0 = male, 1 = female) showed negative associations with Lack of motivation, General indecision, Dysfunctional beliefs, Lack of information about ways to obtain additional information, and External conflicts. School grade (0 = fourth year, 1 = fifth year) exhibited positive associations with all dimensions of career decision-making difficulties except Lack of motivation. Additionally, all career dimensions were found to be correlated with each other.

CFA was performed to assess the factorial structure of CDDQ in this sample of late Italian adolescents. The results reveal a good ten-factor solution with S–Bχ^2^(419) = 1179.301, *p* < 0.001; TLI 0.903; RMSEA 0.049 (0.045–0.052); and SRMR 0.048. All standardized factor loadings were statistically significant and ranged from 0.414 (item 2; Lack of motivation) to 0.873 (item 14; Lack of information about the process).

**Figure 1:**
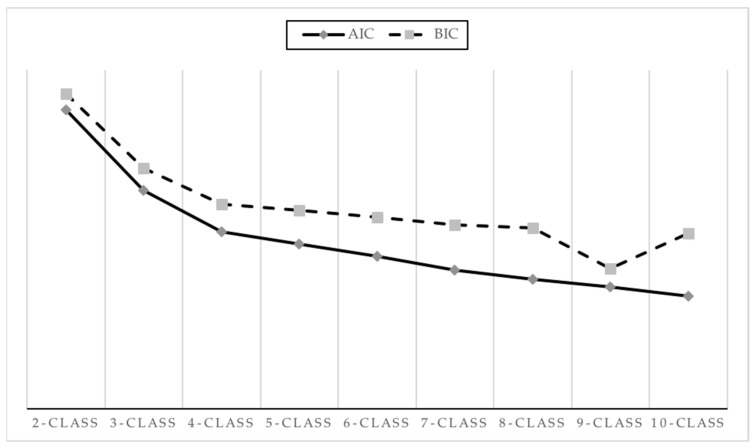
AIC and BIC plot.

**Figure 2:**
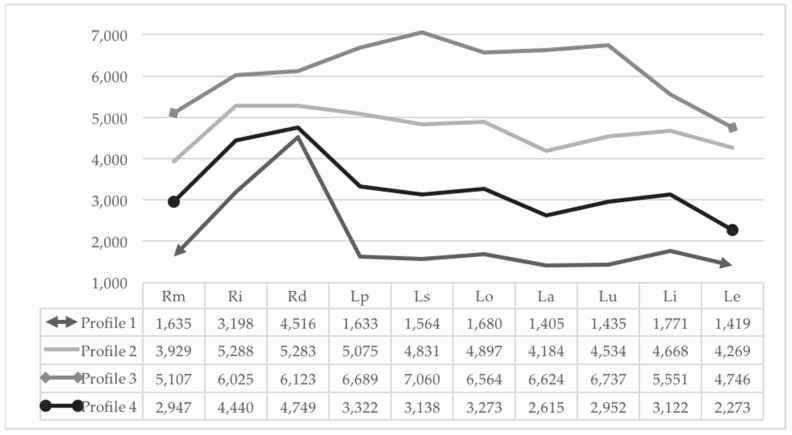
Profiles of career indecision. Note. Rm = Lack of motivation; Ri = General indecisiveness; Rd = Dysfunctional beliefs; Lp = Lack of information about the career decision-making process; Ls = Lack of information about the self; Lo = Lack of information about occupations; La = Lack of information about ways of obtaining additional information; Lu = Unreliable information; Li = Internal conflicts; Le = External conflicts.

LPA was performed to explore profiles of career indecision. The four-class profile, as displayed in Table 3, demonstrated the best fit to the data: low AIC and BIC values (27,565.642 and 27,812.313, respectively), high entropy value (0.895), and significant *p* values for LRT and BLRT. The AIC and BIC plots (Figure 1) demonstrated when the information criteria plateaued, confirming the four-class solution as the optimal choice. The five-profile solution yielded a non-significant *p* value for LRT, indicating that adding another class did not improve model fit. Posterior probabilities for each profile were close to 1 (ranging from 0.916 to 0.960). Profile 1 (39% of the population; N = 300) was labeled the “Lower Indecision”, exhibiting the lowest scores across all career decision-making difficulty dimensions. Profile 2 (23% of the population; N = 182) was labeled “High Indecision”, representing individuals with high levels of career indecision. Profile 3 (7% of the population; N = 53) was labeled “Very High Indecision” as these individuals endorsed the highest scores across all career decision-making difficulties dimensions. Finally, Profile 4 (31% of the population; N = 241) was labeled “Moderate Indecision”, showing moderate levels of general indecision. This four-profile solution is depicted in Figure 2.

The results of the multinomial logistic regression, performed to assess whether school grade and sex predicted profile membership using the “Lower Indecision” (Profile 1) as the reference group, showed no significant differences for sex. Instead, school grade significantly predicted belonging to the High (Profile 2, b = 0.569, SE = 0.242, *p* = 0.019, OR = 1.766, 95%C.I. 1.099–2.838) and Very High Indecision profiles (Profile 3, b = 1.604, SE = 0.327, *p* < 0.001, OR = 4.972, 95%C.I. 2.619–9.439) with older adolescents in the last year of high school demonstrating a higher likelihood of belonging to these profiles.

## 4. Discussions

Utilizing a person-centered approach, this study identified profiles of career indecision using a career decision-making difficulties questionnaire. Results revealed four profiles of career indecision. The first profile exhibited low levels of all career decision-making difficulties and was typified as “Lower Indecision”. This profile includes most of the sample. The second profile, labeled “High Indecision”, displayed high levels of all career decision-making difficulties. The third profile, “Very High Indecision”, comprised adolescents reporting the very highest scores across all career decision-making difficulties dimensions. Despite containing only 7% of the population, this profile represents a high- risk profile. Lastly, the fourth profile, typified as “Moderate Indecision”, was characterized by moderate levels of career indecision and was the second most represented profile among adolescents.

Looking at the shape of the profiles, difficulties related to dysfunctional beliefs, followed by general indecisiveness and lack of motivation, discriminate least among the four profiles, standing relatively high in all profiles. These dimensions, forming the lack of readiness category in Gati’s taxonomy [1], reflect a general state of confusion and a lack of willingness to decide. This finding highlights how the lack of readiness characterizes all profiles from the most to the least indecisive, showing itself as a difficult characteristic for individuals to overcome when they are called upon to make an important choice to continue their studies and choose which university course to take or to look for a job. This is in line with findings in other studies that have shown that this category of difficulty is the least affected by parental support [65] and the most resistant even to career counseling interventions [66,67] probably because it is highly influenced by more stable personality traits [68].

Instead, the lack of information about the self, followed by unreliable information, lack of information about occupations and lack of information about ways of obtaining additional information, is the dimension that differentiates the most among the four profiles. A high score in lack of information about the self indicates a scenario where an individual lacks sufficient information about themselves, including their career preferences and abilities. The dimensions related to the lack of information category achieve the highest levels in Profile 3, “Very High Indecision”, representing a high-risk decision-making profile. This aspect suggests that lack of information about the self and also about the other aspects measured (occupations and how to obtain and discriminate reliable information) are crucial aspects to intervene on with career interventions, both because they particularly characterize the most distressed adolescents and because they can be worked on.

Finally, external conflicts are the decision-making difficulties that differentiate the most the highest from lowest indecision profiles. A high score in this area indicates a gap between individual preferences and those of important others. External factors that might influence career choices are parents [28,29,30,31,32,33,34,35,36,37,38,39,40,41,42,43,44,45,46,47,48,49,50,51,52,53,54,55,56,57,58,59,60,61,62,63,64,65,66,67,68,69]. Previous studies have shown that the most active engagement by the parents should take place during high school to overcome the university vs. ‘work dilemma’, and that perceived parental interference in career choice affects career indecision [28].

Relating to the school grade, findings revealed a significant association between attending the fifth year of high school and a higher probability of belonging to the High and Very High Indecision profiles than were found for the fourth year of high school. This result is in line with previous research supporting that career indecision increases when the individual is close to the career choices [28]. The findings regarding sex did not indicate a significant association between being male or female and the probability of belonging to a specific profile. This result aligns with numerous studies that have similarly not found differences in sex about the career decision-making process [1,3,32,35,36,38,39].

## 5. Limitations and Future Directions

This study has limitations that should be addressed in future research. First, the study uses convenience sampling from late adolescents of southern Italy. The main drawback of this sampling is that the results lack generalizability due to the sample bias. This aspect calls for caution in the interpretation of results and requires planning future studies that may instead allow a generalizability of results. Moreover, the study employs a cross-sectional design, and causal inferences cannot be shown. Future studies should adopt a longitudinal design to determine temporal patterns of profile membership over the career transitions. Second, the measure used, the Career Decision-Making Questionnaire, was self-reported, and consequently, the data may be influenced by a reporting bias. An observational or mixed-methods approach could be used in future studies to provide more objective information about the process of career indecision. Third, no additional substantial data for validation variables were collected to investigate further how the identified profiles differ. An extension of the present study should include additional measures to test predictors and outcomes of profiles. In addition, it would be useful to study which profiles are more likely to be asked for help by a career practitioner and whether the profiles defined as most at risk also present greater psychological health risks (distress) and lower life satisfaction. Furthermore, an analysis of the protective factors associated with the profiles could provide additional information for career guidance practice. Finally, despite the many advantages of the R3STEP method used (see the Statistical Analysis section), R3STEP is limited in its ability to handle missing data, resulting in the list of cases with missing data being deleted. The percentage of missing data is limited in this study (under 5%), so this limitation should not affect the results. Future studies may go in the direction of further investigating the role of sex and school grade.

## 6. Practical Implications

Despite these limitations, this study is the first to examine profiles of career indecision in Italian late adolescents, providing valuable information for career practitioners. This line of research may lead to the development of individualized and more effective career interventions in support of adolescents who are close to career choices. In a traditional approach, Gati and Levin [70] suggested that clients’ CDDQ scores, which are based on the ten dimensions and a global score, could assist career counselors in determining the most suitable interventions. Gati et al. [71] proposed addressing certain difficulties as the highest priority, including lack of motivation, lack of self-information and dysfunctional beliefs, lack of process information, general indecisiveness, and internal and external conflicts. The client can be presented with these difficulties as a selection of potential interventions, allowing them to choose which difficulty to address first with the guidance and support of the career counselor; if working on one difficulty is complicated, he/she can go back to the menu and choose another one. However, both this study and previous research [65,66,67,68] suggest that difficulties associated with lack of readiness (such as lack of motivation, dysfunctional beliefs, and general indecisiveness) may require early interventions with preadolescents, employing a preventive approach, and more intensive interventions with adolescents.

As shown in these typologies, there are no qualitative individual differences in career indecision because there are similar levels between the different aspects of career indecision within each profile. It seems that it is possible to classify the decision-making process of late adolescent students according to two dimensions that could be used as indicators of the whole process of career decision-making in practice: Lack of motivation and Lack of information about the self. Lack of motivation, which refers to the Lack of readiness, is antecedent to the choice process. If motivation is lacking, adolescents perceive themselves as unable to initiate the decision-making process, blocking the initiation of the career choice process. The Lack of information about the self is relevant during the process; therefore, it implies that the decision-making process has begun. The Lack of information about the self refers to feeling capable of facing the choice. This study suggests that adolescents, and in particular those that fit the high and very high profiles of indecision, might particularly need and benefit from interventions focusing on exploring the self that provide insights about how to find a career in line with their identity.

These findings have also implications for policies in Italian secondary schools. In particular, Italian schools do not have career practitioners within the school system. Often, young people turn to psychologists for help or still hope to find answers at the career days offered by Italian universities. However, the spectrum of possibilities is broader than what the adolescent sees and, above all, the employment sector is not represented. If adolescents were more oriented toward the transition to the world of work after high school, they would feel lost and alone. It should be added that the support provided by career days with universities is mostly informative. Instead, it is necessary for the young person to build their own career future by exploring possibilities and cultivating their own motivation. In view of the high level of difficulties, which can be traced back precisely to the lack of readiness, it seems necessary to implement specific career guidance activities in schools in order to work primarily on motivation and the construction of career paths in line with desires and aspirations while at the same time providing (and helping to find) useful information for the process of making career choices in late adolescence. The CDDQ questionnaire proves to be a useful tool to access the ‘world of the adolescent career choices’ and could be a good starting point for school psychologists/practitioners to begin counselling. The more undecided profiles need more work on motivation and self, while the others might benefit from the more informative aspect of the choice.

As suggested by Kulcsár [72], career practitioners can start with a more heterogeneous assessment to diagnose the individual’s problem areas and then use a more homogeneous assessment to understand the specific problem. The CDDQ can be useful as an initial measure to assess the readiness and information categories. Based on the difficulty profile, career practitioners can then decide which homogeneous measure to use to obtain more information about the problem areas.

Concluding, the findings delineate the contributions of the ten career decision-making difficulties to each of the four profiles. The person-centered approach is deemed desirable. Student profiles based on the ten dimensions would be much more helpful to a career counselor than profiles based on the absolute level of career indecision. For example, counseling a student with high motivation and external conflicts would require a different approach than counseling a student with high motivation and lacking information about occupations. This would result in much more differentiated and valuable person-oriented information for counselors than a category based on the level of career indecision alone.

## 7. Conclusions

In summary, this study aims to identify profiles of career indecision in Italian late adolescents. From a person-centered perspective, four profiles seem to exist: “Lower Indecision”, “High Indecision”, “Very High Indecision”, and “Moderate Indecision”. Moreover, these profiles were distinguished by school grade: enrolling in the last year of high school significantly predicted belonging to “High Indecision” and Very High Indecision” profiles. This study has important practical implications, as career practitioners can better identify the risk profiles and develop career interventions with Italian adolescents.

## Figures and Tables

**Table 1:** Means, standard deviations, range, skewness and kurtosis.

	M	SD	Range	Sk	K
Rm	2.814	1.813	1–9	0.837	−0.255
Ri	4.257	2.001	1–9	0.272	−0.652
Rd	4.888	1.735	1–9	0.094	−0.443
Lp	3.307	2.078	1–9	0.684	−0.399
Ls	3.190	1.956	1–9	0.805	−0.191
Lo	3.260	1.905	1–9	0.706	−0.266
La	2.788	1.800	1–9	1.016	0.581
Lu	2.975	1.858	1–9	0.776	−0.233
Li	3.125	1.647	1–9	0.525	−0.599
Le	2.585	1.937	1–9	1.176	0.526

Note. M = mean; SD = standard deviation; Sk = skewness; K = kurtosis; Rm = Lack of motivation; Ri = General indecisiveness; Rd = Dysfunctional beliefs; Lp = Lack of information about the career decision-making process; Ls = Lack of information about the self; Lo = Lack of information about occupations; La = Lack of information about ways of obtaining additional information; Lu = Unreliable information; Li = Internal conflicts; Le = External conflicts.

**Table 2:** Correlation analysis between variables.

	1	2	3	4	5	6	7	8	9	10	11
1. Sex	-										
2. Grade	−0.110 ***	-									
3. Rm	−0.078 **	0.009	-								
4. Ri	−0.170 ***	0.114 ***	0.302 ***	-							
5. Rd	−0.078 **	0.166 ***	0.166 ***	0.168 ***	-						
6. Lp	−0.012	0.168 ***	0.543 ***	0.510 ***	0.203 ***	-					
7. Ls	−0.048	0.153 ***	0.576 ***	0.386 ***	0.170 ***	0.686 ***	-				
8. Lo	−0.054	0.156 ***	0.486 ***	0.384 ***	0.226 ***	0.624 ***	0.702 ***	-			
9. La	−0.089 **	0.179 ***	0.455 ***	0.368 ***	0.208 ***	0.630 ***	0.714 ***	0.729 ***	-		
10. Lu	−0.019	0.154 ***	0.490***	0.423 ***	0.194 ***	0.610 ***	0.682 ***	0.640 ***	0.647 ***	-	
11. Li	−0.069	0.088 **	0.521 ***	0.376 ***	0.211 ***	0.594 ***	0.637 ***	0.648 ***	0.620 ***	0.715 ***	-
12. Le	−0.148 ***	0.180 ***	0.382 ***	0.308 ***	0.259 ***	0.486 ***	0.486 ***	0.507 ***	0.518 ***	0.564 ***	0.626 ***

Note. Rm = Lack of motivation; Ri = General indecisiveness; Rd = Dysfunctional beliefs; Lp = Lack of information about the career decision-making process; Ls = Lack of information about the self; Lo = Lack of information about occupations; La = Lack of information about ways of obtaining additional information; Lu = Unreliable information; Li = Internal conflicts; Le = External conflicts. Sex was coded 0 = male 1 = female. Grade was coded 0 = fourth 1 = fifth. *** *p* < 0.001, ** *p* < 0.01.

**Table 3:** Model comparison.

Fit Statistics	2-Class	3-Class	4-Class	5-Class	6-Class	7-Class	8-Class	9-Class	10-Class
%	64/36	44/34/22	39/23/7/31	31/39/23/ 2/5	36/29/16/ 12/5/2	36/6/16/ 11/25/3/3	35/8/3/10/ 26/4/11/3	5/36/6/3/ 9/21/3/3/14	35/1/9/2/8/ 4/10/3/25/3
AIC	28,647.622	27,934.334	27,565.642	27,459.444	27,350.793	27,230.169	27,150.459	27,080.623	27,001.623
BIC	28,791.900	28,129.808	27,812.313	27,757.310	27,699.955	27,630.427	27,601.912	27,240.319	27,555.467
Entropy	0.924	0.883	0.895	0.908	0.885	0.896	0.894	0.903	0.911
LRT *p* value	<0.001	0.023	<0.001	0.474	0.546	0.216	0.550	0.515	0.773
BLRT *p* value	<0.001	<0.001	<0.001	<0.001	<0.001	<0.001	<0.001	<0.001	<0.001

## Data Availability

The data presented in this study are available upon reasonable request from the corresponding author. The data is not publicly available due to privacy reasons.
